# Gamification in genetics, genomics, and pharmacogenomics education: a bibliometric analysis of research trends, collaboration, and emerging themes (2000–2025)

**DOI:** 10.3389/fmed.2026.1824779

**Published:** 2026-07-07

**Authors:** Azhar T. Rahma, Reem AlSheryani, Noon Hatim Khalid Alrabee, Ahmed Alderei, Alnawa Alharthi, Asma Alshebli, Fatima Alriyami, Sara Alzaabi, Gamila Ahmed, Falah Mohammed AlMarzooqi, Saif Al-Shamsi

**Affiliations:** 1 College of Medicine and Health Sciences, Institute of Public Health, United Arab Emirates University, Al Ain, United Arab Emirates; 2 Public Services and Outreach, National Medical Library, College of Medicine and Health Sciences, United Arab Emirates University, Al Ain, United Arab Emirates; 3 College of Medicine and Health Sciences, United Arab Emirates University, Al Ain, United Arab Emirates; 4 Department of Family Medicine, College of Medicine and Health Sciences, United Arab Emirates University, Al Ain, United Arab Emirates; 5 Department of Internal Medicine, College of Medicine and Health Sciences, United Arab Emirates University, Al Ain, United Arab Emirates

**Keywords:** bibliometric analysis, game-based learning, gamification, genetics, genomics, pharmacogenomics education

## Abstract

The rapid expansion of genomics and pharmacogenomics technologies has created an urgent need for education in genetics, genomics, and pharmacogenomics. Despite their importance in biomedical science and clinical practice, these disciplines remain conceptually challenging to teach and learn, often characterized by low learner engagement and limited conceptual understanding. Gamification, defined as the use of game-based elements to enhance learning experiences, has increasingly been adopted in health and science education to improve motivation, engagement, and skill development. However, evidence describing how gamification has been applied within genomic and pharmacogenomic education remains limited. This study presents a bibliometric analysis of global research trends and thematic developments in gamification applied to genetics, genomics, and pharmacogenomics education between 2000 and 2025. A systematic search of Web of Science, Scopus, and PubMed identified 112 eligible peer-reviewed studies involving gamified educational interventions targeting human learners. Bibliometric analyses were conducted using R and the Bibliometric package to evaluate publication growth, author productivity, institutional affiliations, keyword co-occurrence, and country level collaboration patterns. Results indicate minimal research activity in early years, followed by increased publication growth after 2015. Research output was dominated by institutions in high income countries, with limited contributions from low- and middle-income regions, including the Middle East and North Africa. Thematic analysis revealed emphasis on student engagement, interactive learning, simulation, and digital tools, alongside emerging interest in pharmacogenomics education. Few studies addressed long term learning outcomes. This bibliometric analysis identifies research gaps and informs future educational innovation in genomic and pharmacogenomic education.

**Systematic review registration:**

https://osf.io/5yc9r/overview

## Introduction

Genetics, genomics, and pharmacogenomics are foundational disciplines in biomedical science and clinical practice; their integration into educational curricula has been consistently challenged by the complexity of molecular concepts, the rapid pace of scientific advancement, and persistent deficits in genomic literacy among learners at all educational levels (1–3). Students frequently report difficulty in comprehending abstract genetic mechanisms, interpreting genomic data, and perceiving the clinical or personal relevance of genomic knowledge, particularly when instruction relies on traditional didactic approaches (4, 5). What this field asks of learners goes well beyond factual recall. It demands the ability to reason under uncertainty, interpret complex data, and apply genetic knowledge to clinical decisions (6, 7).

Gamification and game-based learning offer interactive strategies that can support engagement, motivation, conceptual understanding, and applied reasoning (8–10). Educational games and simulations are particularly useful because they allow learners to explore genetic concepts, simplify complex genomic information, and practice decision-making in low-risk learning environments This is what makes gamification and game-based learning worth taking seriously. Rather than asking students to absorb content passively, games place them inside problems they must actively work through, with consequences they can see (2, 11, 12).

Pharmacogenomics is highly relevant to gamification because it requires learners to apply genetic knowledge to real medication decisions, not simply memorize facts. Recent reviews show that active, case-based, simulation-based, and interprofessional pharmacogenomics education can improve knowledge and confidence, but evidence remains fragmented and limited in long-term competence assessment. This supports the need to map how gamification and game-based learning are being used across genetics, genomics, and pharmacogenomics education (13, 14).

This evidence is particularly relevant for countries expanding genomic medicine education and workforce development, including the UAE and the wider MENA region. National genomic initiatives have increased the need for graduates with strong genomic and pharmacogenomic competencies. However, before locally appropriate educational interventions can be developed, it is necessary to understand how gamification and game-based learning in this field have been studied globally. Therefore, this study aims to conduct a comprehensive bibliometric analysis of global research on gamification and game-based learning in genetics, genomics, and pharmacogenomics education.

## Methods

### Study design and registration

This study employed a bibliometric analysis to systematically map the extent, characteristics, and conceptual structure of the scholarly literature examining the use of gamification and game-based approaches in genetics, genomics, and pharmacogenomics education. Bibliometric methods were selected to provide a quantitative and reproducible overview of publication trends, source distribution, authorship patterns, collaboration networks, and thematic development within the field.

The study protocol was prospectively registered as Embargoed registration at the Open Science Framework (OSF) on January 2, 2026.^1^ Protocol registration was undertaken to enhance transparency, methodological rigor, and reproducibility, and to minimize the risk of selective reporting or post hoc analytical decisions.

No deviations from the registered protocol occurred during the conduct of this bibliometric analysis.

### Data sources and search strategy

The librarian-led, multi-database search strategy was designed to maximize sensitivity while maintaining specificity, in line with best practices for bibliometric mapping and PRISMA-ScR recommendations. A comprehensive literature search was conducted across three major bibliographic databases: Web of Science (WoS) Core Collection, Scopus, and PubMed. The search covered publications from January 2000 to December 2025, ensuring capture of both early foundational studies and recent advances in gamification applied to genetics, genomics, and pharmacogenomics education. The final databases search was conducted on 27-December-2025. The search strategy was designed and executed by a professional medical librarian, using a combination of controlled vocabulary [including Medical Subject Headings (MeSH)] and free-text terms searched in titles and abstracts. Keywords and index terms were selected to represent three core conceptual domains:

(1) gamification and game-based learning (e.g., gamification, serious games, educational games, game-based learning, interactive learning);
(2) genetics-related disciplines (e.g., genetics, genomics, pharmacogenomics); and
(3) education and learning outcomes (e.g., education, literacy, knowledge, engagement, skills).

Search strategies were adapted to the indexing structure of each database, and Boolean operators were applied to ensure comprehensive retrieval. The full electronic search strategy for each database is provided in Supplementary material 1.

Search results from all databases were exported and merged, and duplicate records were identified and removed before analysis. The search was limited to English-language publications involving human participants. Consistent with bibliometric methodology, all study designs were eligible, and gray literature was excluded to ensure data quality and consistency.

The initial search identified 291 records from Web of Science, PubMed, and Scopus. After import into Covidence, 111 duplicates were removed, leaving 180 unique records for screening. Following the eligibility assessment, 68 records were excluded, and 112 studies met the inclusion criteria and were included in the final bibliometric analysis.

### Inclusion and exclusion criteria

#### Inclusion criteria

Publications were included in the bibliometric analysis if they met the following criteria:

1. Focused on gamification, game-based learning, or gamified educational interventions applied to genetics, genomics, or pharmacogenomics education.
2. Targeted students or learners at any educational level, including school, undergraduate, postgraduate, or professional training.
3. Addressed educational contexts, learning processes, or learner outcomes related to genomic or pharmacogenomic literacy.
4. Were peer-reviewed journal articles or conference proceedings indexed in Web of Science, Scopus, or PubMed, with complete bibliographic metadata available.
5. Were published in the English language.
6. Involved human participants.
7. Were published within the defined time frame (January 2000–December 2025).

All study designs were eligible for inclusion at this stage, consistent with the objectives of bibliometric mapping.

#### Exclusion criteria

Publication was excluded if they met any of the following criteria:

1. Did not address gamification or game-based approaches within an educational context.
2. Focused exclusively on non-educational applications of genetics, genomics, or pharmacogenomics (e.g., laboratory methods, clinical diagnostics without an educational component).
3. Were non-peer-reviewed sources, including editorials, commentaries, opinion pieces, book chapters, theses, or reports.
4. Were gray literature or not indexed in the selected databases.
5. Were published in languages other than English.
6. Involved non-human subjects.
7. Lacked sufficient bibliographic information to support bibliometric analysis.

In line with PRISMA-ScR guidance, inclusion criteria were intentionally broad to map the extent, range, and nature of the evidence base, without restricting study design or outcome measures at the bibliometric stage.

This review focused exclusively on learners within structured educational environments. Studies involving the general public without a formal educational or training setting were not included. The exclusion criterion for non-human subjects was applied strictly to remove animal and laboratory-based studies without human learners, and not studies involving human learners outside educational contexts.

#### Data extraction and preparation

All records retrieved from Web of Science, Scopus, and PubMed were exported in their original bibliographic formats and merged into a single dataset. Duplicate records were identified and removed using automated matching procedures based on Digital Object Identifiers (DOIs), titles, and author names, followed by manual verification to ensure accuracy.

**FIGURE 1:**
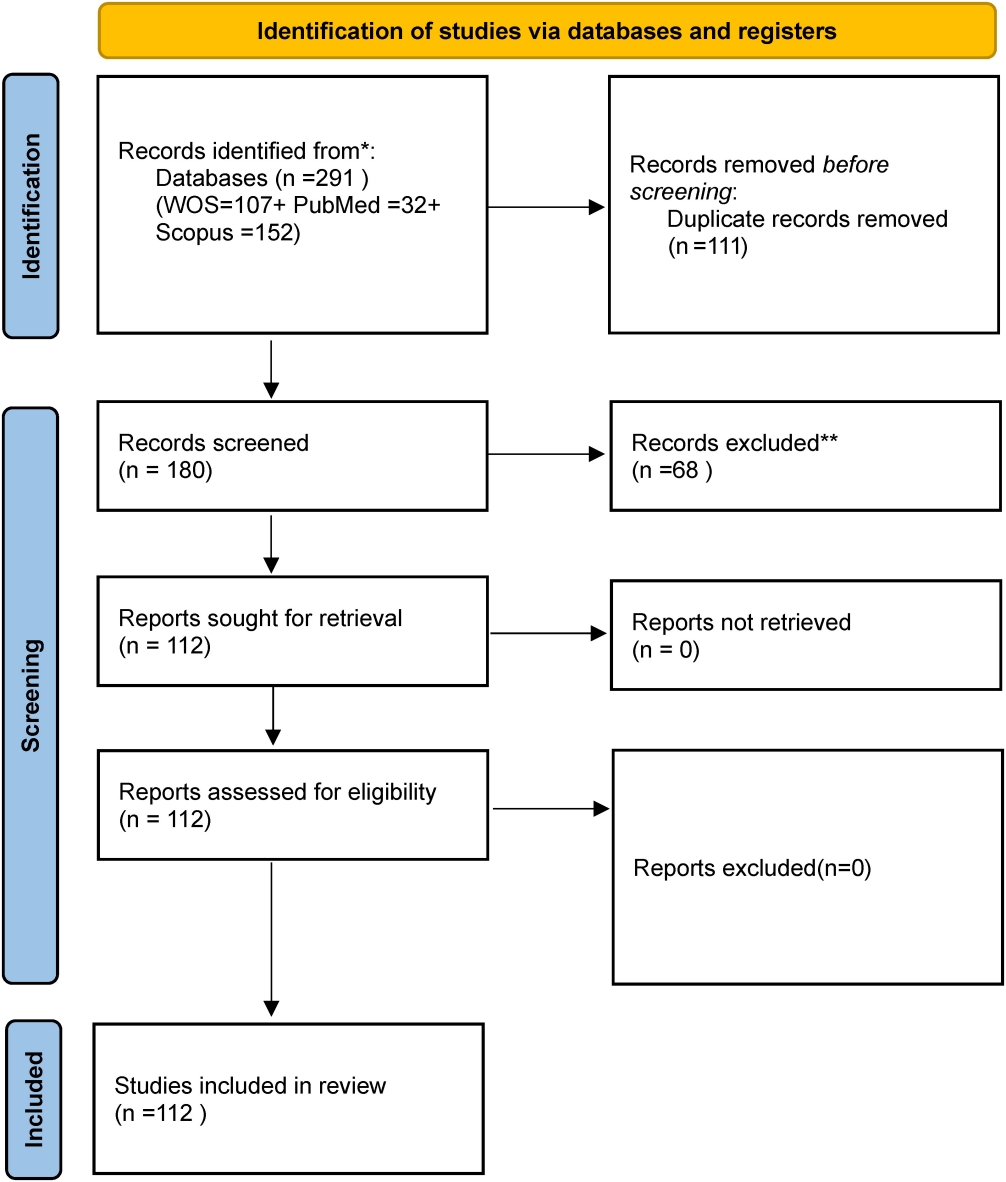
PRISMA flow diagram of studies on gamification in genetics, genomics, and pharmacogenomics education (2000–2025).

Initial record management, including deduplication and screening preparation, was conducted using Covidence systematic review software. Covidence was employed to ensure transparent handling of records and to facilitate the transition from bibliometric mapping to the subsequent scoping review phase. The flow of records through identification, deduplication, and preparation stages is presented in the PRISMA flow diagram (Figure 1).

Bibliographic data extraction was conducted by a professional medical librarian, ensuring consistency and completeness of metadata. Extracted variables included publication year, document type, author names, corresponding author information, institutional affiliations, country of origin, source titles, citation counts, and keywords (both author-provided and database-indexed).

A structured data cleaning and harmonization process was applied before analysis. Author keywords and indexed keywords were merged into a single unified keyword field to reduce redundancy and enhance conceptual consistency. Synonyms, spelling variations, and plural forms were standardized (e.g., “game-based learning” and “game-based learning”), and non-informative terms were removed. Records with missing keyword information were retained to avoid systematic exclusion, with such entries coded as “NA” where applicable.

Country and institutional affiliations were standardized to address variations in naming conventions across databases. For country-level productivity analyses, the corresponding author’s country was used. Collaboration patterns were categorized as single-country publications (SCP) or multiple-country publications (MCP).

#### Bibliometric analysis

Bibliometric analyses were conducted using R statistical software (version 4.5.2), primarily through the Bibliometrix package and its web-based interface Bibliophagy. These tools were selected due to their robustness, transparency, and widespread use in science mapping and bibliometric research.

##### Descriptive bibliometric indicators

Descriptive analyses were performed to examine annual scientific production, document types, citation patterns, and publication growth over time. These indicators were used to characterize the temporal evolution of research on gamification in genetics, genomics, and pharmacogenomics education.

##### Source and author analysis

Journal productivity and source dispersion were assessed using Bradford’s Law of Scattering to identify core journals contributing disproportionately to the literature. Sources were ranked by number of publications and divided into Bradford zones, distinguishing a core set of highly productive journals from secondary and peripheral sources. This analysis was used to characterize publication concentration and to identify the principal outlets shaping the field.

##### Author productivity and Lotka’s Law

Author productivity patterns were examined using Lotka’s Law, allowing assessment of the distribution of scholarly output across authors. This analysis facilitated identification of prolific contributors and characterization of the core–periphery structure of authorship within the field.

##### Citation and impact analysis

Citation-based indicators, including total citations and source-level impact metrics, were calculated to describe the relative influence of journals and publications within the dataset. Citation analysis was used descriptively to map influence rather than to infer study quality or effectiveness.

##### Keyword and co-word analysis

Keyword frequency analysis was conducted using both author keywords and indexed keywords, merged during data preparation. Co-word analysis was performed to identify conceptual relationships among keywords and to map the field’s intellectual structure. Network clustering was conducted using the walk trap algorithm, which identifies groups of closely connected terms based on random walks within the keyword network.

##### Thematic mapping and trend analysis

Thematic maps were generated based on centrality and density measures, classifying themes into motor, basic, niche, and emerging or declining categories. Temporal trend analysis was used to identify keywords and themes that increased in prominence over time, highlighting shifts in research focus and the emergence of new pedagogical and technological approaches.

##### Collaboration analysis

Country-level collaboration networks were constructed using corresponding author affiliation data. Publications were categorized as single-country publications (SCP) or multiple-country publications (MCP) to examine patterns of international collaboration. Network visualizations and centrality measures were used to describe the structure and connectivity of global research collaborations.

##### Methodological considerations

Consistent with PRISMA-ScR principles, bibliometric analyses were used to map the extent, range, and characteristics of the evidence base. No critical appraisal of individual studies was undertaken at this stage, as the objective was descriptive mapping rather than evaluation of educational effectiveness. Findings from this bibliometric analysis were intended to inform the design and scope of a subsequent scoping review.

All bibliometric indicators were applied descriptively and interpreted within the limitations of citation-based analyses, without inferring causality or educational effectiveness.

### Ethical considerations

This study involved secondary analysis of publicly available bibliographic data retrieved from Web of Science, Scopus, and PubMed. No human participants, personal data, or identifiable information were involved. As such, ethical approval and informed consent were not required.

All data were accessed through institutional subscriptions and handled in accordance with database terms of use. The study protocol was registered on the Open Science Framework, supporting transparency and responsible research conduct. Reporting was informed by PRISMA-ScR principles, and no ethical concerns related to participant risk or data confidentiality were identified.

## Results

### Publication trend (annual scientific production)

**FIGURE 2:**
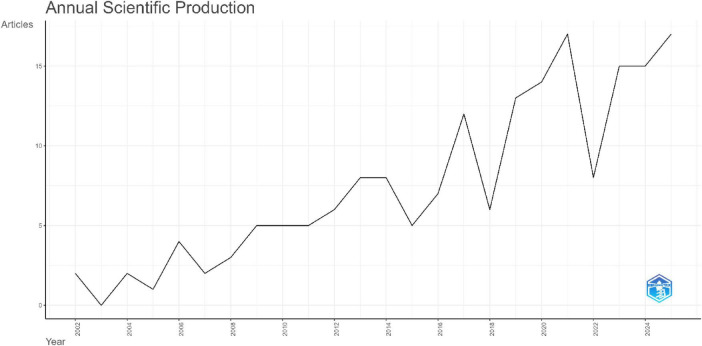
Trends in annual scientific production related to gamification in genetics and genomics education (2000–2025).

The analysis included publications indexed between 2000 and 2025. Annual scientific production demonstrated a clear upward trajectory over time, with minimal research output observed during the early years of the study period. From 2000 to approximately 2010, publications were sporadic, with fewer than two articles per year, indicating limited early scholarly attention to gamification in genetics and genomics education within the study timeframe (2000–2025) (Figure 2).

A gradual increase in publication volume was observed from 2011 onwards, followed by a marked acceleration after 2015. This period corresponds to the broader adoption of digital learning technologies and increased interest in innovative pedagogical approaches within science and medical education. The most substantial growth occurred between 2018 and 2024, during which the majority of included publications were produced, reflecting intensified research activity and consolidation of the field.

Peak publication Production Related to Gamification in Genetics and Genomics Education was recorded in the early 2020s, with annual production reaching its highest levels between 2020 and 2024. This surge coincided with increased global emphasis on technology-enhanced learning and curriculum innovation, particularly in response to remote and blended education models.

Overall, the annual scientific production pattern indicates a transition from an emerging research area to a rapidly expanding and increasingly established field, characterized by sustained growth in scholarly output between 2000 and 2025.

**FIGURE 3:**
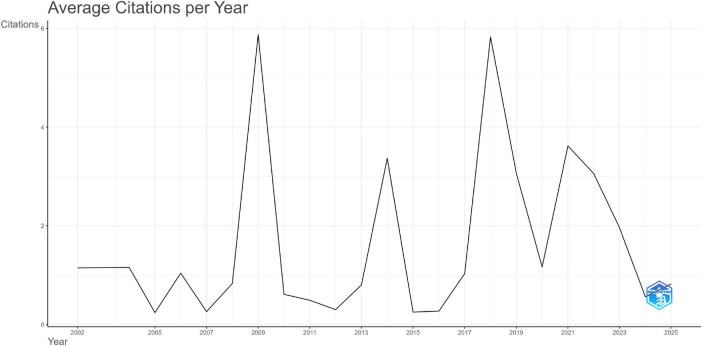
Average citations per article per year in gamification research on genetics, genomics, and pharmacogenomics education (2000–2025).

In addition to growth in publication volume, citation impact exhibited notable temporal variation over the study period (Figure 3). Average citations per year remained relatively low and stable during the early years of the field, generally below one citation per article, reflecting the limited size and visibility of early publications. A pronounced citation peak was observed around 2009, when average citations reached approximately six citations per article, followed by a sharp decline in subsequent years. Smaller but distinct citation increases were noted around 2014 and again between 2018 and 2019, indicating periods during which highly cited publications emerged. “This citation peak is attributable to a very small number of publications indexed in 2009, specifically, a highly cited study by Gorbanev et al. on serious games in medical education, which accrued disproportionate citations relative to the limited annual output. This artifact is consistent with the statistical behavior of citation-based metrics in emerging fields, where individual influential papers can dominate average citation rates when annual publication counts are fewer than five. Accordingly, the 2009 spike should be interpreted as reflecting the impact of isolated landmark publications rather than a sustained period of high scholarly activity.”

Following 2020, average citations per year showed moderate fluctuation, with values ranging between approximately 1 and 4 citations per article, before declining again in the most recent years. The lower citation averages observed after 2023 likely reflect citation lag, as recently published articles have had limited time to accrue citations. Overall, the citation pattern complements the observed increase in publication volume, suggesting that growth in research output has been accompanied by intermittent periods of higher scholarly impact rather than a uniform increase in citation intensity.

Together, annual scientific production and citation trends indicate a field characterized by accelerating output alongside episodic peaks in citation impact

#### Source distribution and core journals

Analysis of source distribution revealed a highly uneven concentration of publications across journals, consistent with Bradford’s Law of Scattering. A relatively small number of journals accounted for a substantial proportion of the total literature, while the majority of sources contributed only a limited number of publications.

**FIGURE 4:**
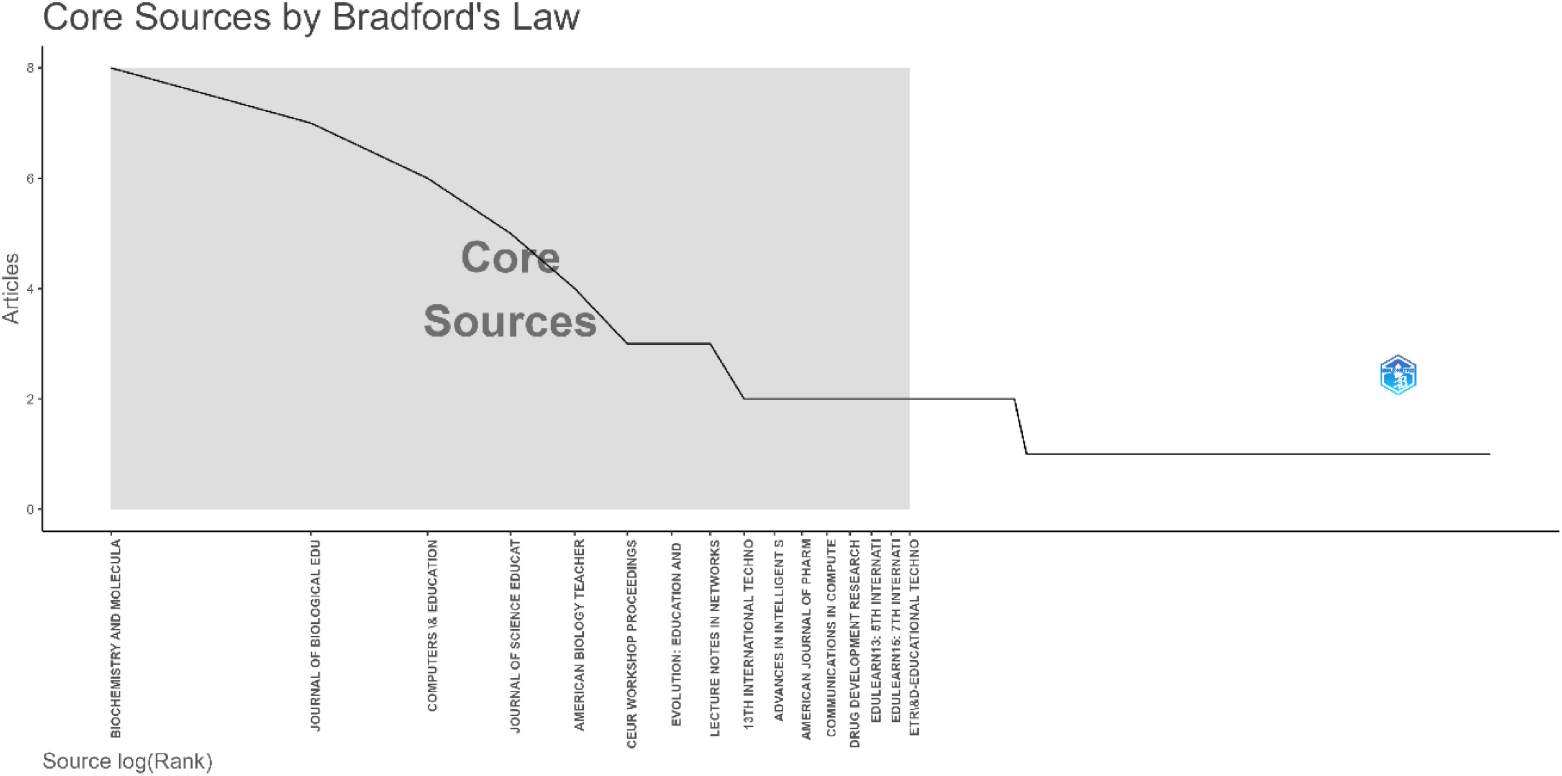
Core journals identified by Bradford’s Law in gamification research on genetics, genomics, and pharmacogenomics education (2000–2025).

The core Bradford zone (Figure 4) comprised a small group of education- and technology-focused journals that each published between 6 and 8 articles on gamification in genetics, genomics, and pharmacogenomics education. The most productive and locally influential sources included *Computers and Education* (H-index = 6), *Journal of Biological Education* (H-index = 5), and *Journal of Science Education and Technology* (H-index = 5). Additional core outlets included *Biochemistry and Molecular Biology Education* (H-index = 4) and *American Biology Teacher* (H-index = 2), reflecting strong representation of biology and science education journals within the field.

Beyond the core zone, publications were dispersed across a large number of secondary and peripheral journals, most of which contributed one or two articles only. These sources included a mix of interdisciplinary education journals, conference proceedings, and technology-oriented outlets. This long tail of low-productivity sources reflects the interdisciplinary nature of the field, spanning education, life sciences, and educational technology.

Overall, the Bradford distribution demonstrates that while research on gamification in genetics and genomics education is disseminated across a wide range of outlets, scholarly communication remains anchored in a small set of core journals that shape the development and visibility of the field.

The observed Bradfordian scattering pattern confirms the presence of a stable core of journals alongside broad dissemination across peripheral sources.

#### Author productivity (leading contributor and productivity analysis)

**FIGURE 5:**
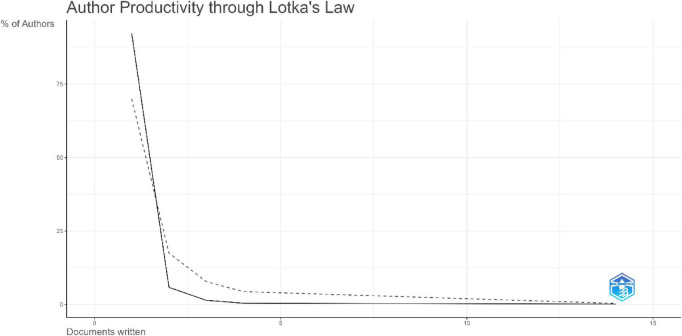
Author productivity pattern based on Lotka’s Law in gamification research on genetics, genomics, and pharmacogenomics education (2000–2025).

Author productivity analysis revealed a highly skewed distribution of scholarly output, consistent with Lotka’s Law (Figure 5), whereby a small number of authors contributed multiple publications while the majority authored a single article.

Most authors in the dataset produced one publication only, indicating a broad and dispersed authorship base. In contrast, a limited group of authors emerged as leading contributors, demonstrating sustained research activity over time. These leading authors each contributed between two and three publications, representing the most productive individuals within the field.

Temporal analysis of author productivity showed that leading contributors were primarily active during the mid-2010s to early-2020s, coinciding with the period of accelerated publication growth observed in annual scientific production. Several authors demonstrated intermittent publication patterns, with outputs clustered around specific years rather than continuous annual productivity, suggesting project- or intervention-based research engagement.

Citation intensity varied among productive authors. While some leading contributors accrued higher total citations and citations per year, others demonstrated moderate citation impact despite multiple publications, reflecting heterogeneity in article visibility and influence.

Overall, the observed authorship structure indicates that research on gamification in genetics, genomics, and pharmacogenomics education is characterized by a core–periphery pattern, with a small group of recurring contributors supporting field development alongside a large number of occasional authors. This pattern reflects the interdisciplinary and emerging nature of the field, attracting contributors from education, life sciences, and educational technology backgrounds.

The Lotka-type productivity distribution indicates a developing research area with limited author consolidation and broad participation across disciplines.

### Global and institutional contributions

**FIGURE 6:**
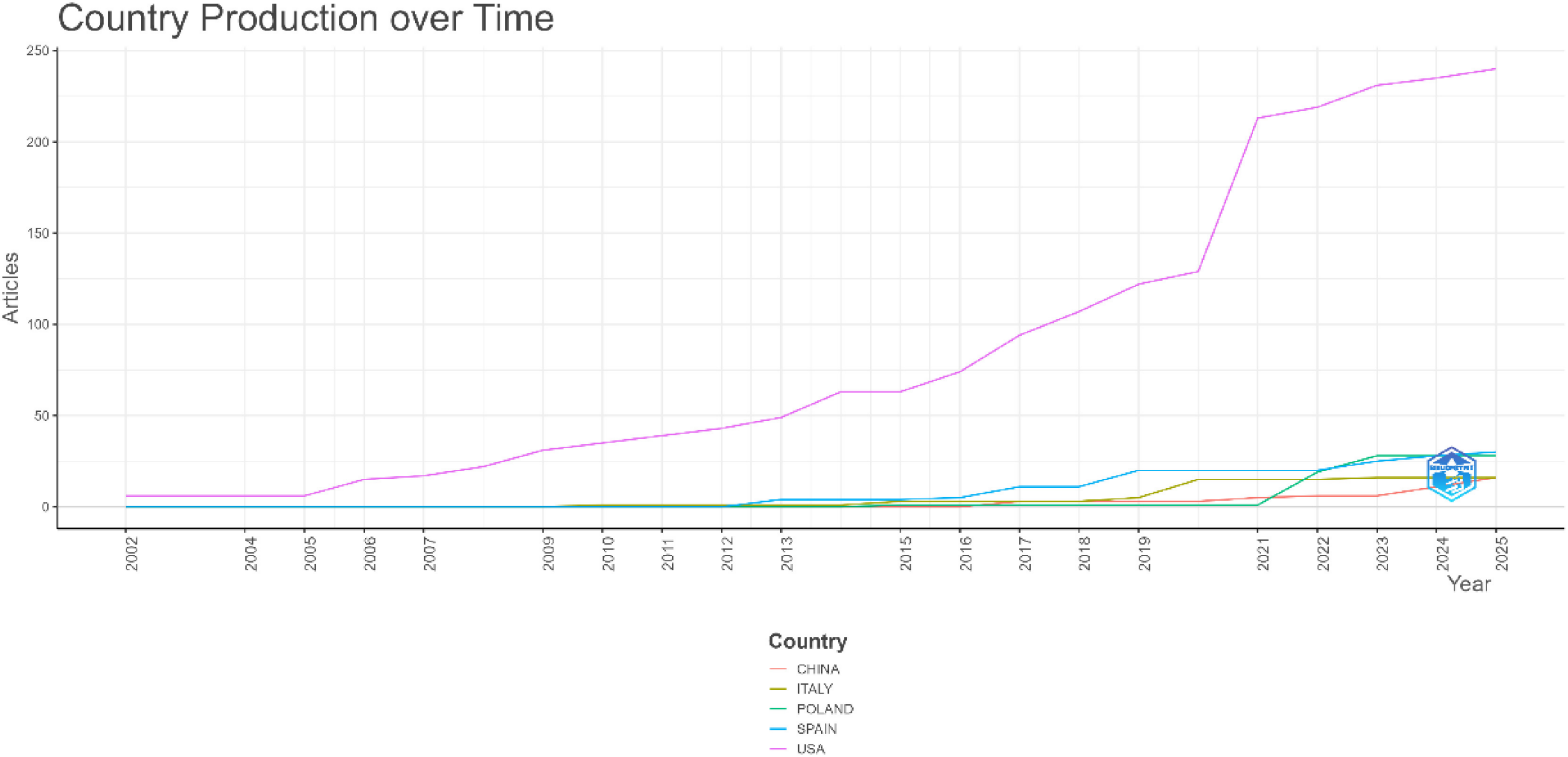
Country production trends over time in gamification research on genetics, genomics, and pharmacogenomics education (2000–2025).

**FIGURE 7:**
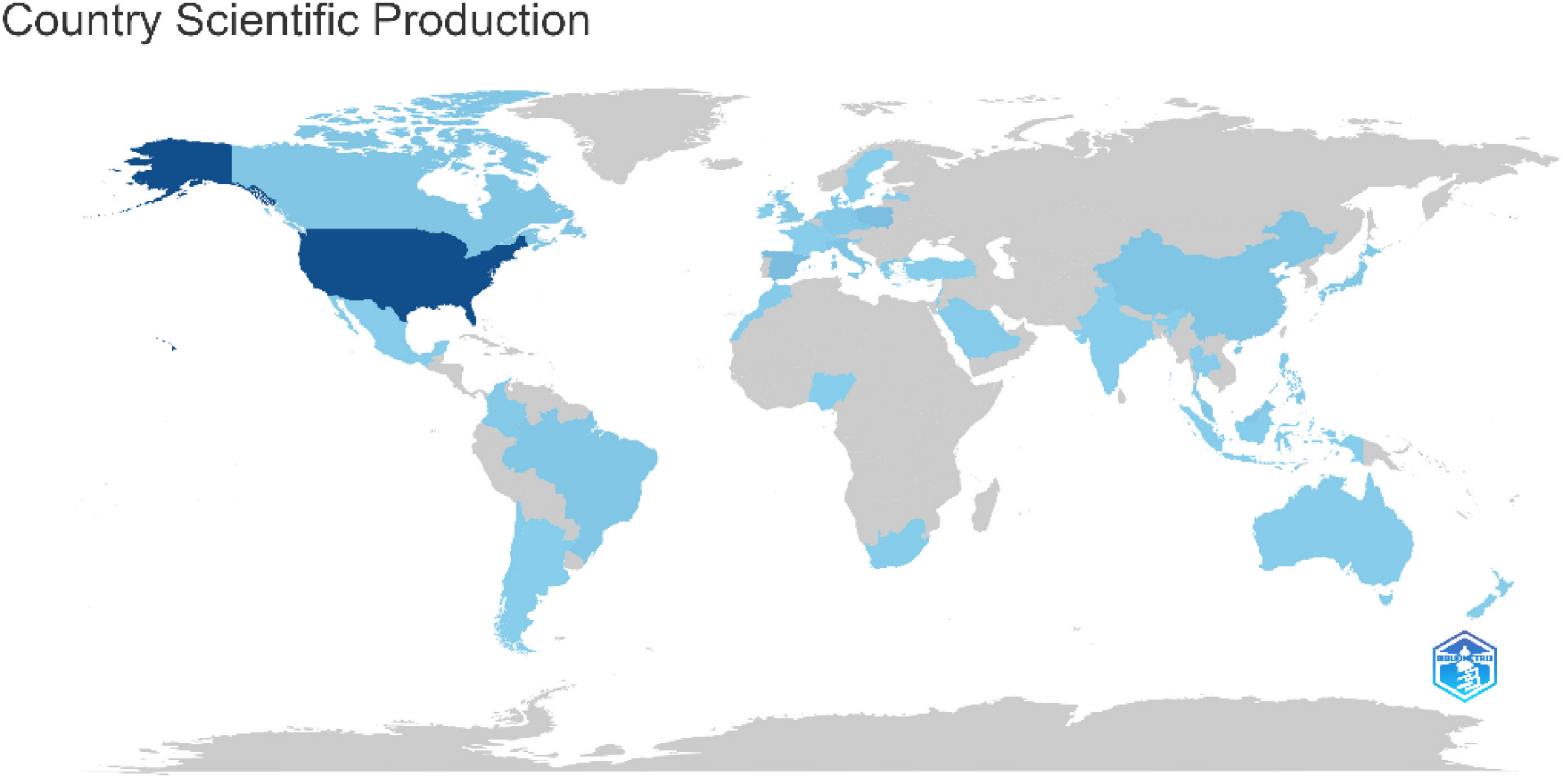
Worldwide scientific output by country in gamification research on genetics, genomics, and pharmacogenomics education (2000–2025).

The global distribution of research on gamification in genetics and genomics education demonstrates a geographically diverse yet uneven pattern of scientific contribution, with a strong concentration in a limited number of high-income countries (Figures 6, 7). In total, corresponding authors were affiliated with institutions across more than 30 countries, reflecting the growing international relevance of this interdisciplinary field.

#### Country-level scientific production

The United States emerged as the dominant contributor, accounting for approximately 50 documents, representing nearly half of the total corpus. Of these, the majority were single-country publications (SCPs), indicating strong domestic research capacity, while a smaller but meaningful proportion were multiple-country publications (MCPs), underscoring its central role in international collaboration networks.

Following the United States, China ranked second with approximately 9 documents, of which a notable share involved international collaboration (MCPs), reflecting increasing cross-border engagement in educational innovation research. Spain (≈8 documents), Brazil (≈7 documents), and Canada (≈7 documents) also contributed substantially, with Canada and several European countries demonstrating a higher proportion of MCPs relative to SCPs, suggesting a more collaborative research profile.

Countries such as Italy, Colombia, Malaysia, and the United Kingdom each contributed between 4 and 6 documents, predominantly through SCPs, while Denmark, Germany, Japan, Mexico, the Philippines, Thailand, Chile, India, the Netherlands, and Poland contributed smaller but consistent outputs, typically ranging from 2 to 4 documents per country.

#### Single-country vs. multiple-country publications

Overall, the dataset was characterized by a predominance of single-country publications, accounting for approximately 75–80% of all documents, while multiple-country publications represented around 20–25% of the total output. This imbalance indicates that, although international collaboration exists, the field remains largely driven by national research initiatives, particularly in leading countries such as the United States and China.

**FIGURE 8:**
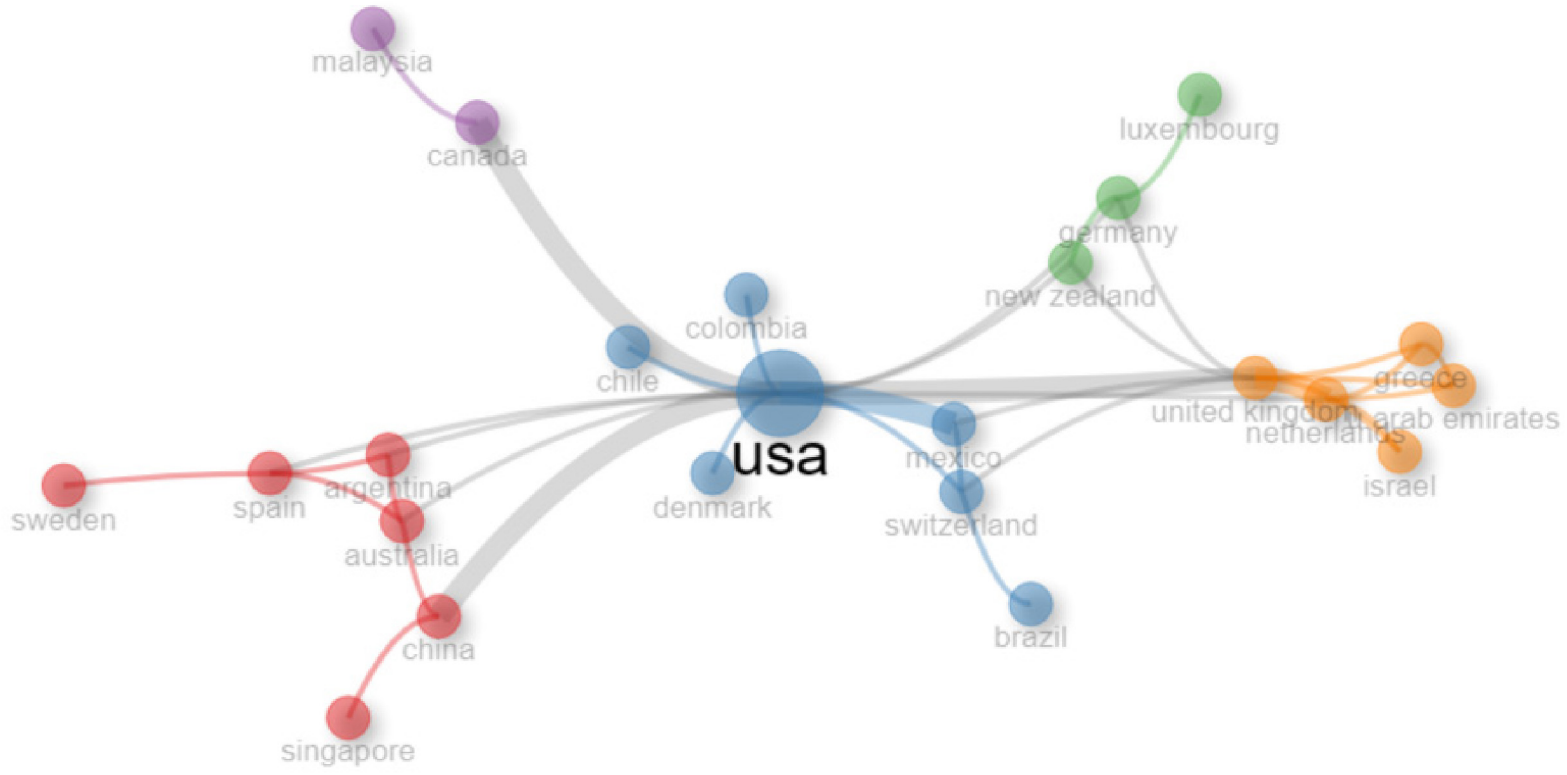
Global country collaboration network in gamification research on genetics, genomics, and pharmacogenomics education (2000–2025).

However, the collaboration network analysis (Figure 8) highlights several strong bilateral and multilateral partnerships, notably between:

- the United States and European countries (e.g., United Kingdom, Germany, Netherlands),
- the United States and Latin American countries (e.g., Brazil, Colombia, Mexico),
- and emerging Asia–Pacific collaborations involving China, Singapore, Malaysia, and Australia.

These collaborative links are visually represented by thicker network edges, indicating higher co-authorship frequency and stronger collaborative intensity.

#### Institutional and regional patterns

At the institutional level, highly productive countries tended to host central nodes within the collaboration network, reinforcing their role as knowledge hubs. Institutions based in the United States frequently acted as bridging institutions, connecting otherwise weakly linked regions and facilitating knowledge transfer across continents.

Regionally, North America and Europe dominated scientific production, while Asia showed rapid growth over time, particularly after 2015, consistent with the upward trend in annual scientific output. Contributions from Africa and parts of the Middle East remained limited, highlighting ongoing regional disparities in research capacity within this domain.

Collectively, these findings indicate that research on gamification in genetics and genomics education is globally distributed but structurally centralized, with a small number of countries accounting for a disproportionate share of output and collaboration leadership. The dominance of SCPs suggests opportunities for expanding international, multicenter research efforts, particularly as the field moves toward more applied and evaluative phases, including the planned scoping review.

#### Keyword frequency analysis

Keyword frequency analysis was conducted to identify the dominant concepts, pedagogical approaches, and disciplinary foci within the included literature. A total of over 450 author keywords were extracted and standardized through manual cleaning to merge synonyms and harmonize spelling variants before analysis.

**FIGURE 9:**
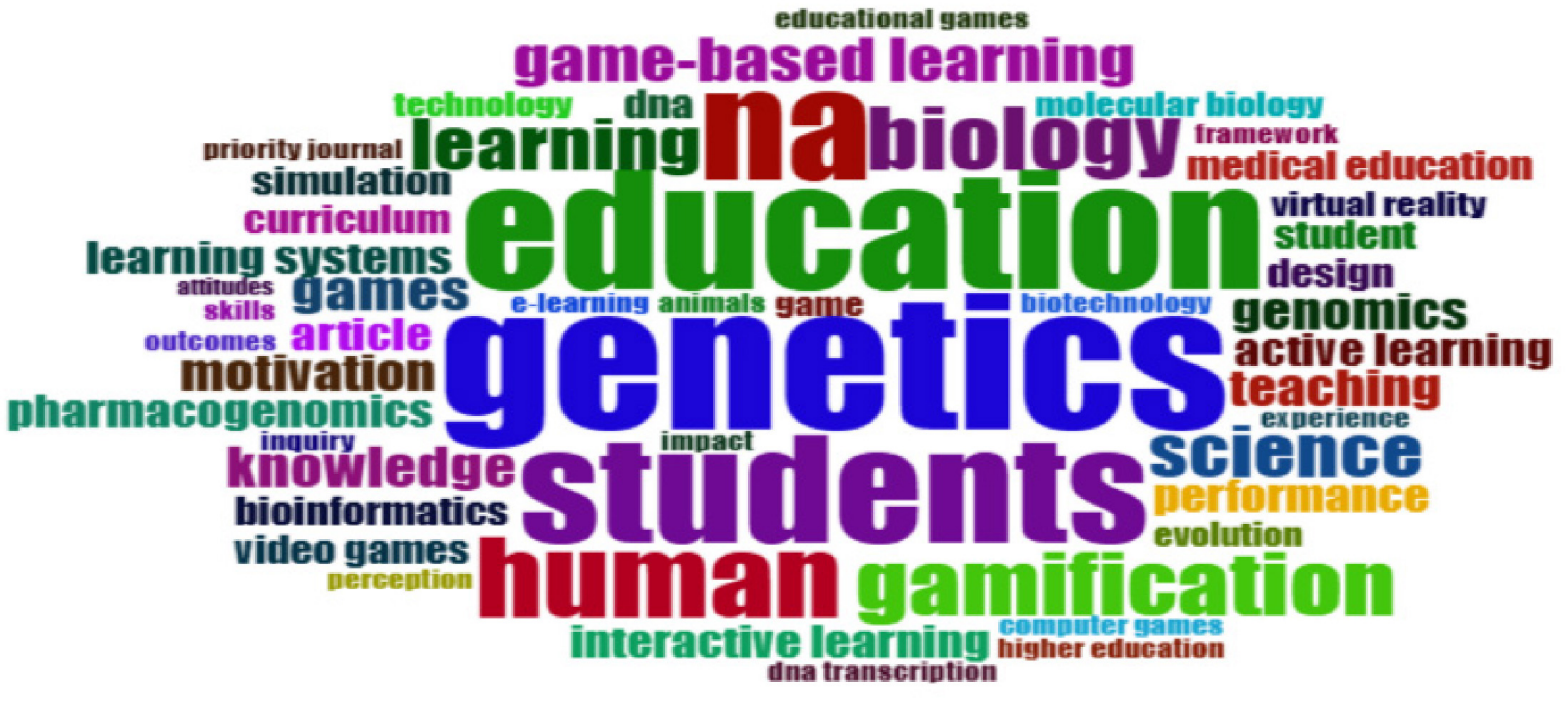
Word cloud of author keyword frequency in gamification and genetics education research (2000–2025).

The most frequently occurring keyword was “genetics” (*n* = 34; ∼7% of total keyword occurrences), followed by “education” (*n* = 30; ∼6%) and “students” (*n* = 27; ∼6%). These findings confirm that the literature is primarily centered on educational interventions targeting learners within genetics-related domains. The keyword “human” also appeared prominently (*n* = 20; ∼4%), indicating a strong emphasis on human-focused educational contexts rather than model organisms or purely theoretical content (Figure 9).

Pedagogical and instructional strategy–related keywords were highly prevalent. “Gamification” (*n* = 16; ∼3%) and “game-based learning” (*n* = 11; ∼2%) emerged as central instructional approaches, alongside “games” (*n* = 10; ∼2%), “simulation” (*n* = 7; ∼2%), and “interactive learning” (*n* = 8; ∼2%). Additional learning-related terms such as “active learning” (*n* = 8; ∼2%), “motivation” (*n* = 9; ∼2%), “performance” (*n* = 8; ∼2%), and “knowledge” (*n* = 10; ∼2%) highlight the outcome-oriented focus of many studies.

Disciplinary keywords reflected a strong representation of molecular and genomic sciences. “Biology” (*n* = 16; ∼3%), “genomics” (*n* = 9; ∼2%), “DNA” (*n* = 7; ∼2%), “molecular biology” (*n* = 6; ∼1%), “bioinformatics” (*n* = 7; ∼2%), and “pharmacogenomics” (*n* = 8; ∼2%) indicate that gamification strategies have been applied across multiple subdomains of genetics and genomics education. The presence of “medical education” (*n* = 7; ∼2%) further suggests growing integration of these approaches within professional and clinical training contexts.

Technology-enhanced learning modalities were also evident, with keywords such as “e-learning” (*n* = 5; ∼1%), “learning systems” (*n* = 8; ∼2%), “technology” (*n* = 6; ∼1%), and “virtual reality” (*n* = 6; ∼1%). These findings point to increasing experimentation with digital and immersive tools alongside traditional game-based methods.

#### Co-occurrence analysis

**FIGURE 10:**
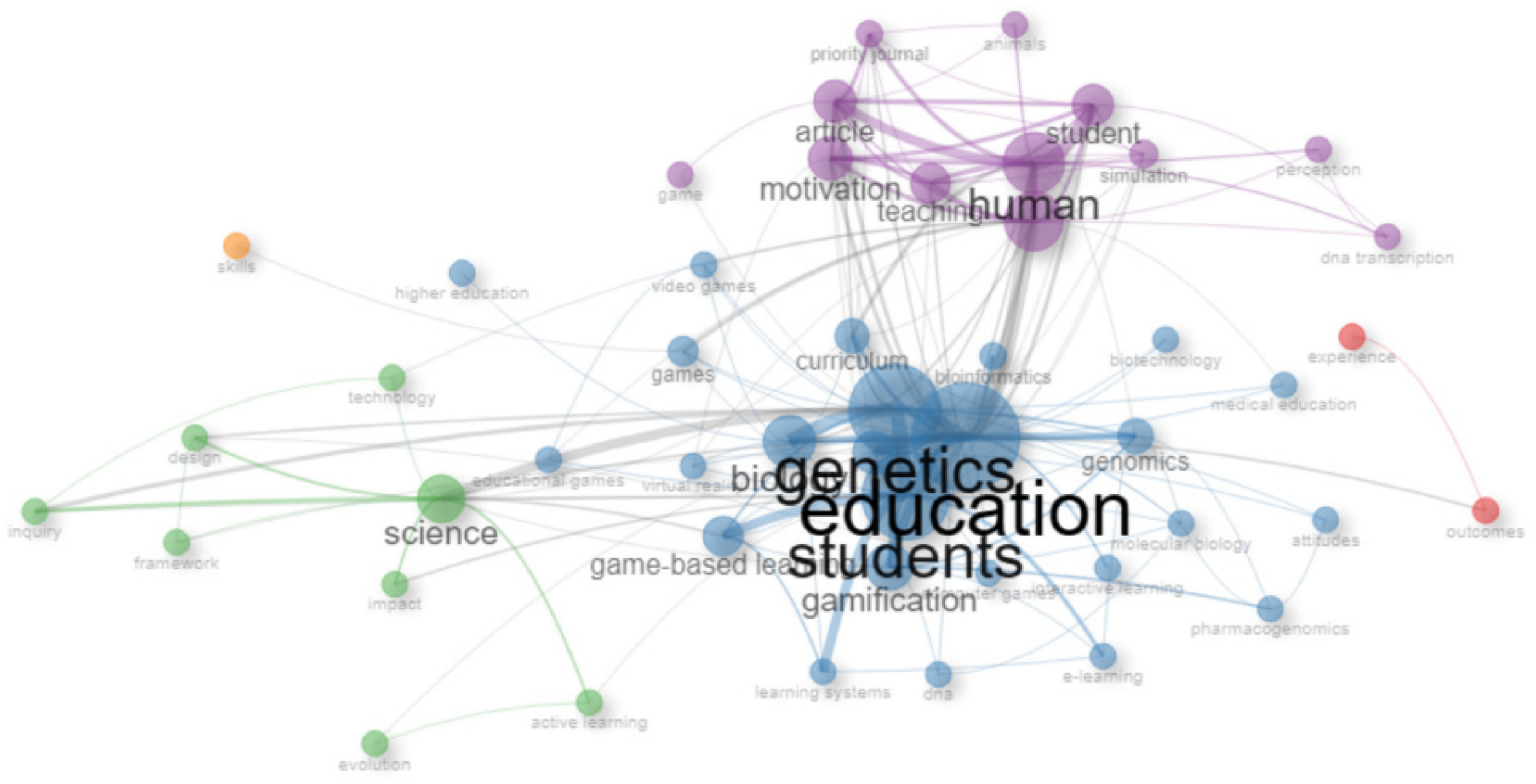
Keyword co-occurrence network in gamification and genetics education research (2000–2025).

The co-occurrence analysis of author keywords reveals the conceptual structure of the literature by identifying frequently associated terms (Figure 10). Prominent clusters center around genetics, education, students, and gamification, indicating a strong thematic linkage between educational strategies and genetics-related learning outcomes. Closely connected terms such as *game-based learning*, *active learning*, *simulation*, and *virtual reality* highlight the growing integration of interactive and digital pedagogies. The presence of domain-specific keywords, including *genomics*, *pharmacogenomics*, *molecular biology*, and *medical education*, reflects the multidisciplinary application of gamification approaches across biomedical and health education contexts. The walk trap clustering algorithm identified four principal keyword clusters within the co-occurrence network. Cluster 1, anchored by “genetics,” “education,” and “students,” represents the consolidated core of the field, topics with high centrality and sustained presence throughout the study period, indicative of a mature research team. Cluster 2, organized around “gamification,” “game-based learning,” and “Motivation,” constitutes an established but still actively developing area, with increasing keyword density after 2015. Cluster 3, comprising “virtual reality,” “Simulation,” and “digital tools,” represents an emerging theme characterized by growing co-occurrence frequency in recent years (2018–2024), suggesting a field in transition toward immersive technologies. Cluster 4, encompassing “pharmacogenomics,” “precision medicine,” and “clinical education,” represents a niche but strategically important theme with low density but high centrality, indicating relevance to the field’s development without yet having attracted a critical mass of research. Topics such as “long-term outcomes” and “implementation science” were conspicuously absent from all clusters, confirming the critical gap in evaluative research identified in the discussion.

## Discussion

This bibliometric analysis identified four key patterns: a rapid increase in publications after 2015; strong concentration of research in high-income countries with limited international collaboration; a thematic focus on engagement and interactive learning with limited attention to long-term outcomes; and clear underrepresentation of low- and middle-income countries, including the MENA region.

Temporal trends show a sharp rise in publication output after 2015, consistent with the broader growth of gamification research in medical and health professions education (15). However, our study shows that the genetics, genomics, and pharmacogenomics subset remains much narrower and less consolidated.

The analysis of source distribution shows a small number of journals contributed a relatively higher number of articles. The most productive journals, including *Computers and Education* and *Biochemistry and Molecular Biology Education*, each contributed a limited number of publications (16). Publications beyond the core journals were scattered across a wide range of disciplinary outlets, a pattern consistent with bibliometric evidence from gamification in health education, where studies have been distributed across hundreds of distinct journals (17). The spread of publications across diverse and disciplinarily distinct journals may hinder the consolidation of evidence and reduce the broader applicability of findings within genetics, genomics, and pharmacogenomics education.

Author productivity patterns, consistent with Lotka’s Law, reveal a concentration of scholarly output among a small cohort of recurring contributors, with the episodic nature of many authorship patterns suggesting project-based rather than programmatic research engagement, which limits the diversity of educational contexts represented in the literature. This concentration of output within a small number of recurring contributors may limit the diversity of educational contexts and pedagogical approaches represented in the literature. Broader institutional participation and more rigorous methodological protocols are needed to strengthen evidence-based educational innovation (18, 19).

Thematic co-occurrence analysis highlights the conceptual evolution of the field toward active learning, simulation, and immersive technologies. Yet the limited presence of keywords related to educational outcomes and implementation of science underscores a crucial disconnect between pedagogical design and evaluative reliability, same pattern noticed in prior meta-analyses of gamified learning (9). Specifically, few studies report on long-term knowledge retention, transfer of learning to clinical practice, or behavioral change, and fewer still examine institutional adoption, cost-effectiveness, or sustainability.

The emergence of virtual reality, simulation, and immersive digital tools as a distinct and growing thematic cluster in the keyword co-occurrence analysis signals an important directional shift in the field, reflecting broader trends in health professions education toward technology-enhanced and experiential learning environments. However, the bibliometric evidence suggests this remains an early-stage development within genetics and genomics education specifically, with limited evaluative evidence to date on whether immersive technologies produce superior learning outcomes compared to conventional game-based approaches (20).

Regionally, contributions from the MENA region were limited despite growing genomic initiatives. Abou Tayoun et al. documented that fragmented services, scarce training programs, and persistent brain drain directly constrain educational research capacity, compounded further by well-documented publishing inequities including insufficient writing support, editorial underrepresentation, and bias against researchers from economically challenged areas. The dominance of English, accounting for 86.5% of PubMed-indexed publications, renders Arabic-language scholarship effectively invisible to international bibliometric databases. These intersecting structural inequities present a timely opportunity for regionally led research networks, open-access publishing support, and contextually adapted gamified interventions co-designed with MENA educators (21, 22).

While bibliometric analysis offers a structured overview of research trends, it does not evaluate the quality, design, or contextual nuance of individual studies. Reliance on keyword-based co-occurrence networks may overlook thematic subtleties or interdisciplinary overlaps not captured in metadata. Insights from the bibliometric analysis, particularly thematic clusters, trend topics, and geographic distribution, will guide future scoping review. to provide a comprehensive overview of both the structure of the literature and the nature of empirical evidence, supporting evidence-informed educational practice and future research directions.

## Limitations

Several limitations should be acknowledged. First, the total corpus of 112 eligible publications, while the most comprehensive identified to date for this specific intersection of gamification and genetics/genomics/ pharmacogenomics education, is relatively modest for a bibliometric analysis spanning 25 years. This reflects the genuinely emerging nature of the field rather than a methodological limitation per se; however, it constrains the robustness of certain inferences. Specifically, claims regarding the “intellectual structure” and “global collaboration patterns” of the field should be interpreted as preliminary mappings rather than definitive structural characterizations. The very low annual publication counts during 2000–2010 (fewer than 2 publications per year) are particularly susceptible to distortion in citation-based metrics, as a single highly-cited article can disproportionately inflate average citation figures for that period. Moreover, the analysis is dependent on the selected database and indexing practices, which may result in the omission of relevant publications not captured in the dataset. Additionally, bibliometric indicators primarily reflect research productivity and connectivity, rather than study quality or educational effectiveness. Moreover, keyword-based analyses may be influenced by inconsistencies in terminology across studies. Additionally, only studies published in English were included, which may have excluded relevant research published in other languages. Finally, as a bibliometric study, the findings are descriptive rather than evaluative, underscoring the need for the planned scoping review to provide deeper contextual and qualitative insights.

## Strengths of the study

This study employs a rigorous and transparent bibliometric methodology to map the development, structure, and thematic evolution of research on gamification in genetics and genomics education. The use of validated analytical tools (R and the *bibliometrix* package), combined with librarian-led data extraction, enhances data accuracy and reproducibility. Integration of multiple bibliometric indicators—including publication trends, core journals, author productivity, collaboration networks, and keyword co-occurrence—provides a comprehensive and multidimensional overview of the field. Protocol registration on OSF and alignment with PRISMA-ScR principles further strengthen methodological credibility and support open science practices.

## Conclusion

This bibliometric analysis provides a comprehensive overview of the research landscape on gamification and game-based learning in genetics, genomics, and pharmacogenomics education. The findings demonstrate a steady growth in scientific output over time, with scholarly activity concentrated in a small number of core journals and led by a limited group of recurring contributors, alongside a broad base of occasional authors. Thematic and keyword analyses highlight a strong emphasis on learner-centered, technology-enhanced educational approaches, with increasing attention to advanced genomic concepts and innovative pedagogical strategies.

Despite the expanding and increasingly international nature of the field, research production and collaboration remain unevenly distributed, with a predominance of single-country publications and limited cross-national partnerships. These patterns suggest opportunities to strengthen global collaboration and diversify institutional participation.

Overall, this study maps the extent, structure, and evolution of the evidence base and identifies key gaps related to effectiveness, implementation, and educational outcomes. The findings provide a robust foundation for a subsequent PRISMA-ScR–guided scoping review, which will synthesize empirical evidence to inform future research, educational practice, and curriculum development in genetics and genomics education.

## Data Availability

The original contributions presented in this study are included in the article/Supplementary material, further inquiries can be directed to the corresponding author.

## Footnotes

1
https://osf.io/ve7aw/overview
