# Supplementary material for: Gamification in genetics, genomics, and pharmacogenomics education: a bibliometric analysis of research trends, collaboration, and emerging themes (2000–2025)
Source: Front Med (Lausanne). 2026 Jul 7;13:1824779. doi: 10.3389/fmed.2026.1824779 (PMC13386216; doi:10.3389/fmed.2026.1824779)
Supplement: Supplementary file 1 [file Table_1.docx]

**Supplementary material**

| **Supplementary Material 1.** A detailed description of the search terms and strategy. | | | | |
| --- | --- | --- | --- | --- |
| **Source and coverage** | **Search string** | | **Results** | **notes** |
| PubMed (NLM)  Coverage:  From database inception-2024-01-28 | (((((((((((((((("Simulation-Based Learning "[Title/Abstract]) ) OR ("Simulation Training"[Title/Abstract])) OR ("Medical Simulation"[Title/Abstract])) OR ("Simulation-Based Education"[Title/Abstract])) OR ("Interactive Training"[Title/Abstract])) OR ("Virtual Reality (VR)"[Title/Abstract])) OR ("VR-Based Learning "[Title/Abstract])) OR ("Immersive Learning"[Title/Abstract])) OR ("Augmented Reality (AR) "[Title/Abstract])) OR ("AR-Based Training"[Title/Abstract])) OR ("High-Fidelity Simulation "[Title/Abstract])) OR ("Low-Fidelity Simulation "[Title/Abstract])) OR ("Mixed Reality (MR) "[Title/Abstract])) OR ("Experiential Learning"[Title/Abstract])) AND (((((((((((((("Medical Education"[Title/Abstract]) OR ("Undergraduate Medical Education "[Title/Abstract])) OR ("Graduate Medical Education "[Title/Abstract])) OR ("Continuing Medical Education"[Title/Abstract])) OR ("Medical Training "[Title/Abstract])) OR ("Medical Teaching"[Title/Abstract])) OR ("Clinical Skills Training "[Title/Abstract])) OR ("Interprofessional Education (IPE) "[Title/Abstract])) OR ("Area Health Education Centers "[Title/Abstract])) OR ("Fellowships[Title/Abstract] AND Scholarships "[Title/Abstract])) OR ("Internship and Residency"[Title/Abstract])) OR ("Public Health Professional Education "[Title/Abstract])) OR ("Health Professions Education "[Title/Abstract])) OR ("Faculty Development"[Title/Abstract]))) AND ((((((("Educational Technology"[Title/Abstract]) OR ("Computer-Assisted Instruction"[Title/Abstract])) OR ("Manikins) "[Title/Abstract])) OR ("Technology-Enhanced Education "[Title/Abstract])) OR ("Technology-Enhanced Learning"[Title/Abstract])) OR ("AI"[Title/Abstract])) OR ("Artificial Intelligence"[Title/Abstract])) Filters: Bibliography, Case Reports, Clinical Conference, Clinical Study, Clinical Trial, Clinical Trial, Phase I, Clinical Trial, Phase II, Clinical Trial, Phase III, Clinical Trial, Phase IV, Clinical Trial Protocol, Consensus Development Conference, Consensus Development Conference, NIH, Controlled Clinical Trial, Observational Study, Practice Guideline, Preprint, Randomized Controlled Trial, Review, Scientific Integrity Review, Systematic Review, Technical Report, Validation Study, English, from 2000 - 2025 | | [34](https://pubmed.ncbi.nlm.nih.gov/?term=longqueryef89068ba11d7913fed3&filter=dates.2000-2025&filter=pubt.bibliography&filter=pubt.casereports&filter=pubt.clinicalconference&filter=pubt.clinicalstudy&filter=pubt.clinicaltrial&filter=pubt.clinicaltrialphasei&filter=pubt.clinicaltrialphaseii&filter=pubt.clinicaltrialphaseiii&filter=pubt.clinicaltrialphaseiv&filter=pubt.clinicaltrialprotocol&filter=pubt.consensusdevelopmentconference&filter=pubt.consensusdevelopmentconferencenih&filter=pubt.controlledclinicaltrial&filter=pubt.observationalstudy&filter=pubt.practiceguideline&filter=pubt.preprint&filter=pubt.randomizedcontrolledtrial&filter=pubt.review&filter=pubt.scientificintegrityreview&filter=pubt.systematicreview&filter=pubt.technicalreport&filter=pubt.validationstudy&filter=lang.english&sort=relevance) | All search terms are searched in the field: [Title/Abstract] and in MeSH (when available). filters or English language, articles and reviews  Years 2000-2025 |
| Scopus (Elsevier)  Coverage:  From database inception-2024-01-28 | ( TITLE-ABS-KEY ( "Simulation-Based Learning" ) OR TITLE-ABS-KEY ( "Simulation Training" ) OR TITLE-ABS-KEY ( "Medical Simulation" ) OR TITLE-ABS-KEY ( "Simulation-Based Education" ) OR TITLE-ABS-KEY ( "Interactive Training" ) OR TITLE-ABS-KEY ( "Virtual Reality (VR)" ) OR TITLE-ABS-KEY ( "VR-Based Learning" ) OR TITLE-ABS-KEY ( "Immersive Learning" ) OR TITLE-ABS-KEY ( "Augmented Reality (AR)" ) OR TITLE-ABS-KEY ( "AR-Based Training " ) OR TITLE-ABS-KEY ( "Low-Fidelity Simulation" ) OR TITLE-ABS-KEY ( "High-Fidelity Simulation" ) OR TITLE-ABS-KEY ( "Mixed Reality (MR)" ) OR TITLE-ABS-KEY ( "Experiential Learning" ) ) AND ( TITLE-ABS-KEY ( "Medical Education " ) OR TITLE-ABS-KEY ( "Undergraduate Medical Education " ) OR TITLE-ABS-KEY ( "Graduate Medical Education " ) OR TITLE-ABS-KEY ( "Continuing Medical Education " ) OR TITLE-ABS-KEY ( "Medical Training" ) OR TITLE-ABS-KEY ( "Medical Teaching " ) OR TITLE-ABS-KEY ( "Clinical Skills Training " ) OR TITLE-ABS-KEY ( "Interprofessional Education (IPE) " ) OR TITLE-ABS-KEY ( "Area Health Education Centers " ) OR TITLE-ABS-KEY ( "Fellowships and Scholarships " ) OR TITLE-ABS-KEY ( "Internship and Residency " ) OR TITLE-ABS-KEY ( "Public Health Professional Education" ) OR TITLE-ABS-KEY ( "Health Professions Education " ) OR TITLE-ABS-KEY ( "Faculty Development" ) ) AND ( TITLE-ABS-KEY ( "Educational Technology " ) OR TITLE-ABS-KEY ( "Computer-Assisted Instruction" ) OR TITLE-ABS-KEY ( "Manikins for medical training" ) OR TITLE-ABS-KEY ( "Technology-Enhanced Education " ) OR TITLE-ABS-KEY ( "Technology-Enhanced Learning " ) OR TITLE-ABS-KEY ( "AI" ) OR TITLE-ABS-KEY ( "Artificial Intelligence" ) ) AND PUBYEAR > 1999 AND PUBYEAR < 2026 AND ( LIMIT-TO ( DOCTYPE , "ar" ) OR LIMIT-TO ( DOCTYPE , "re" ) ) AND ( LIMIT-TO ( LANGUAGE , "English" ) ) | | 480 results | All search terms are searched in the fields: “title”, “abstract” and “keywords” (here marked with “TITLE-ABS-KEY”) filters or limitations English language, articles and reviews  Years 2000-2025 |
| Web of Science-Core Collection (Clarivate)  Coverage:  From database inception-2024-01-28 | #1 (((((((((((((TS=("Simulation-Based Learning" )) OR TS=(" Simulation Training ")) OR TS=("Medical Simulation" )) OR TS=("Simulation-Based Education" )) OR TS=("Interactive Training ")) OR TS=("Virtual Reality (VR)")) OR TS=("VR-Based Learning ")) OR TS=("Immersive Learning" )) OR TS=("Augmented Reality (AR) ")) OR TS=("AR-Based Training" )) OR TS=("High-Fidelity Simulation" )) OR TS=("Low-Fidelity Simulation ")) OR TS=("Mixed Reality (MR)" )) OR TS=("Experiential Learning")  <https://www.webofscience.com/wos/woscc/summary/a2d4c0e2-9c76-4b9a-8743-973cc6e1debd-014c88c8eb/relevance/1>  #2(((((((((((((TS=("Medical Education " )) OR TS=(" Undergraduate Medical Education ")) OR TS=("Graduate Medical Education " )) OR TS=("Continuing Medical Education " )) OR TS=(" Medical Training")) OR TS=("Health Professions Education ")) OR TS=("Public Health Professional Education ")) OR TS=("Faculty Development " )) OR TS=("Medical Teaching ")) OR TS=("Clinical Skills Training " )) OR TS=(" Interprofessional Education (IPE)" )) OR TS=("Area Health Education Centers ")) OR TS=("Fellowships and Scholarships " )) OR TS=("Internship and Residency ")  <https://www.webofscience.com/wos/woscc/summary/32bdb06e-3306-452f-9dbb-90696557adae-014c88da81/relevance/1>  #3 ((((((TS=("Educational Technology ")) OR TS=("Computer-Assisted Instruction" )) OR TS=("Manikins for medical training" )) OR TS=("Technology-Enhanced Education ")) OR TS=("Technology-Enhanced Learning ")) OR TS=("AI")) OR TS=("Artificial Intelligence")  <https://www.webofscience.com/wos/woscc/summary/02266145-0890-489b-a738-ee5ad05b44d4-014ca3f57f/relevance/1>  #1&#2&#3  <https://www.webofscience.com/wos/woscc/summary/4160e21a-cf2d-4a1b-89ed-2966a232aaab-014ca4782b/relevance/1> | | [99](https://www-webofscience-com.uaeu.idm.oclc.org/wos/woscc/summary/4160e21a-cf2d-4a1b-89ed-2966a232aaab-014ca4782b/relevance/1) | All search terms are searched in the field: “Topic” (including title, abstract and author supplied keywords, here marked with “TOPIC”). filters or limitations English language, articles and reviews  Years 2000-2025 |
| Total no. references identified | | 613 |  |  |
| Total no. unique references identified after automatic de-duplication in Covidence | | 520 |  |  |
| duplicate | | 93 |  |  |

| **Supplementary Material 2**. Sources Local impact by H Index |
| --- |
| 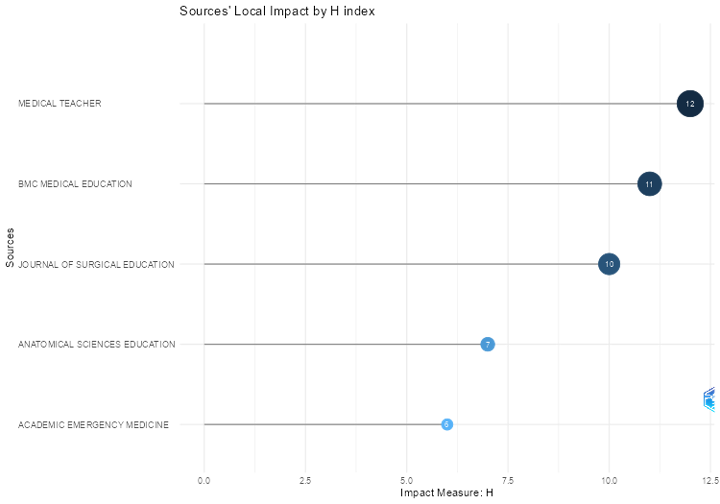 |

| **Supplementary Material 3.** The top three influential authors in medical simulation research | | | |
| --- | --- | --- | --- |
| Authors | year | Title | Journal |
| SCHIJVEN M | 2021 | Development of a model for video-assisted postoperative team debriefing | Journal of surgical research |
|  | 2015 | Porcine cadaver organ or virtual-reality simulation training for laparoscopic cholecystectomy: a randomized, controlled trial | Journal of surgical education |
|  | 2014 | Augmented reality in medical education? | Perspectives on medical education |
|  | 2012 | Designing a proficiency-based, content validated virtual reality curriculum for laparoscopic colorectal surgery: a delphi approach | surgery |
|  | 2009 | Serious gaming and voluntary laparoscopic skills training: a multicenter study | Minimally invasive therapy and allied technologies |
|  | 2009 | The value of haptic feedback in conventional and robot-assisted minimal invasive surgery and virtual reality training: a current review | Surgical endoscopy |
|  | 2005 | The eindhoven laparoscopic cholecystectomy training course - improving operating room performance using virtual reality training: results from the first e.a.e.s. accredited virtual reality trainings curriculum | Surgical endoscopy and other interventional techniques |
| COOK D A | 2025 | Creating virtual patients using large language models: scalable, global, and low cost | Medical teacher |
|  | 2020 | Adaptive instruction and learner interactivity in online learning: a randomized trial | Advances in health sciences education |
|  | 2018 | Educational technologies for physician continuous professional development: a national survey | Academic medicine |
|  | 2013 | simulation training for breast and pelvic physical examination: a systematic review and meta-analysis | Bjog: an international journal of obstetrics and gynaecology |
|  | 2012 | Comparative effectiveness of technology-enhanced simulation versus other instructional methods a systematic review and meta-analysis | Simulation in healthcare-journal of the society for simulation in healthcare |
|  | 2012 | Mastery learning simulation-based curriculum for laparoscopic tep inguinal hernia repair | Journal of surgical education |
| AGGARWAL R | 2012 | Recent advancements in medical simulation: patient-specific virtual reality simulation | World journal of surgery |
|  | 2011 | Training in surgical oncology - the role of vr simulation | Surgical oncology |
|  | 2010 | Training and simulation for patient safety. | Quality & safety in health care |
|  | 2008 | Training opportunities and the role of virtual reality simulation in acquisition of basic laparoscopic skills | Journal of surgical research |
|  | 2007 | Proving the effectiveness of virtual reality simulation for training in laparoscopic surgery | Annals of surgery |

| **Supplementary material 4.** Most Relevant affiliations |
| --- |
| 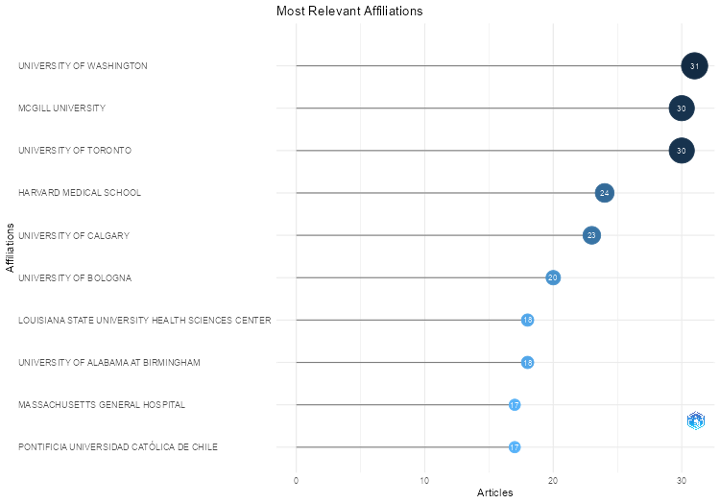 |

| **Supplementary material 5.** Most relevant affiliation of simulation-based learning literature | | |
| --- | --- | --- |
|  | **Affiliation** | **Articles** |
|  | University of Washington | 31 |
|  | McGill University | 30 |
|  | University of Toronto | 30 |
|  | Harvard Medical School | 24 |
|  | University of Calgary | 23 |
|  | University of Bologna | 20 |
|  | Louisiana state University Health Sciences Center | 18 |
|  | University of Alabama at Birmingham | 18 |
|  | Massachusetts general hospital | 17 |
|  | Pontificia Universidad Católica de Chile | 17 |
|  | Imperial College London | 16 |
|  | Mayo Clinic College of Medicine | 16 |
|  | Mayo Clinic | 14 |
|  | University of California | 13 |
|  | University of New Mexico | 13 |
|  | Brigham and Women's Hospital | 12 |
|  | National University of Singapore | 12 |
|  | University of Pittsburgh Medical Center | 12 |
|  | Brown University | 11 |
|  | University of Erlangen-Nuernberg | 11 |
|  | Université de Paris | 11 |
|  | Virginia Commonwealth University | 11 |
|  | Zhejiang University | 11 |
|  | Kyushu University | 10 |
|  | Lund University hospital | 10 |
|  | New York University Grossman School of Medicine | 10 |
|  | Stanford University School of Medicine | 10 |
|  | Univ Pecs | 10 |
|  | University Health Network | 10 |
|  | University of Minnesota | 10 |

| **Supplementary material 6.** Most Global Cited Documents |
| --- |
| 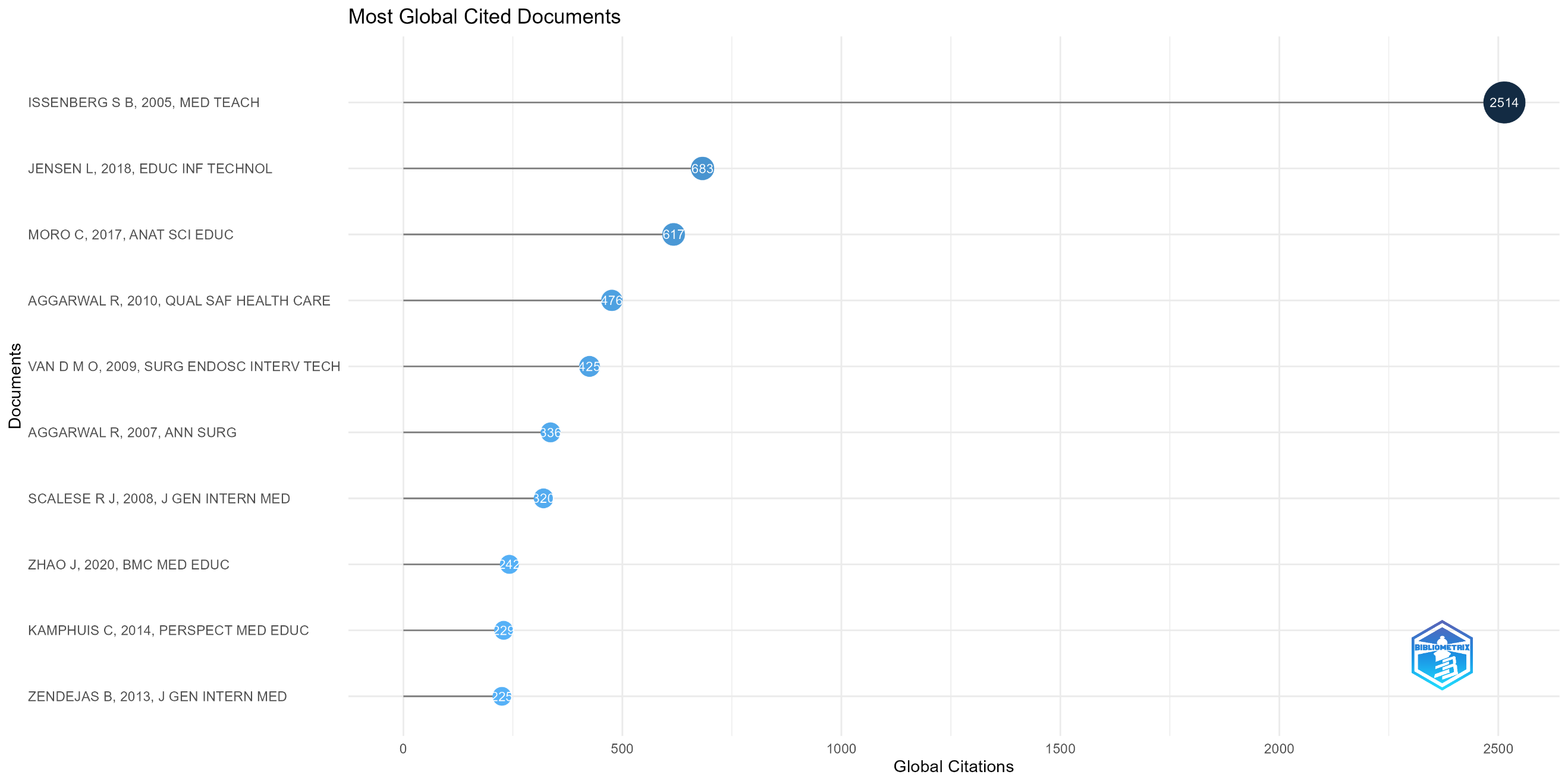 |

| **Supplementary material 7.** Co-occurrence network of the key words in medical simulation research |
| --- |
| 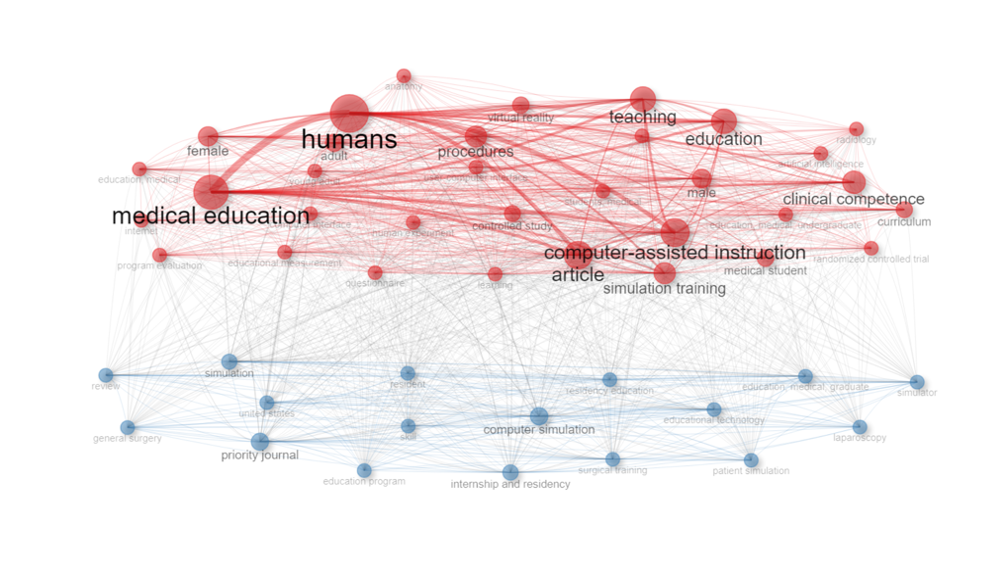 |
